# Routine phasing of coiled-coil protein crystal structures with *AMPLE*

**DOI:** 10.1107/S2052252515002080

**Published:** 2015-02-26

**Authors:** Jens M. H. Thomas, Ronan M. Keegan, Jaclyn Bibby, Martyn D. Winn, Olga Mayans, Daniel J. Rigden

**Affiliations:** a Institute of Integrative Biology, University of Liverpool, Liverpool L69 7ZB, England; b Research Complex at Harwell, STFC Rutherford Appleton Laboratory, Didcot OX11 0FA, England; c Science and Technology Facilities Council, Daresbury Laboratory, Warrington WA4 4AD, England

**Keywords:** molecular replacement, *ab initio* modelling, coiled-coil proteins, search-model ensembles, macromolecular complexes

## Abstract

Coiled-coil protein folds are among the most abundant in nature. These folds consist of long wound α-helices and are architecturally simple, but paradoxically their crystallographic structures are notoriously difficult to solve with molecular-replacement techniques. The program *AMPLE* can solve crystal structures by molecular replacement using *ab initio* search models in the absence of an existent homologous protein structure. *AMPLE* has been benchmarked on a large and diverse test set of coiled-coil crystal structures and has been found to solve 80% of all cases. Successes included structures with chain lengths of up to 253 residues and resolutions down to 2.9 Å, considerably extending the limits on size and resolution that are typically tractable by *ab initio* methodologies. The structures of two macromolecular complexes, one including DNA, were also successfully solved using their coiled-coil components. It is demonstrated that both the *ab initio* modelling and the use of ensemble search models contribute to the success of *AMPLE* by comparison with phasing attempts using single structures or ideal polyalanine helices. These successes suggest that molecular replacement with *AMPLE* should be the method of choice for the crystallo­graphic elucidation of a coiled-coil structure. Furthermore, *AMPLE* may be able to exploit the presence of a coiled coil in a complex to provide a convenient route for phasing.

## Abstract 2

*AMPLE* solved 80% of a large set of coiled-coil protein targets of diverse architectures by molecular replacement with *ab initio* structure predictions. Successes included targets of up to 253 residues, cases of diffraction to only 2.9 Å resolution and macromolecular complexes containing proteins with other folds or DNA.

## 1. Introduction   

The coiled coil is a simple protein architecture that mediates the self-association and hetero-association of proteins into functional quaternary assemblies. Coiled coils consist of a number of amphipathic α-helices, typically two to five, wound around each other to form a supercoil. This distinct fold is achieved by protein sequences consisting of characteristic seven-residue or 11-residue repeats, which lead to left-handed or right-handed coiling, respectively (Lupas & Gruber, 2005 ▶). Coiled-coil folds are found ubiquitously in nature and adopt a variety of sizes and oligomeric states. Their scaffolding function underlies many fundamental processes in biology, including transcription, ATP synthesis, intracellular transport, transmembrane signalling, membrane fusion and remodelling, proteostasis, the formation of the extracellular matrix and several cytoskeletal and nuclear structures of the eukaryotic cell (see, for example, Baxevanis & Vinson, 1993 ▶; Kuhn *et al.*, 2014 ▶). Accordingly, mutations of coiled-coil proteins have been associated with significant human diseases such as progeria (Broers *et al.*, 2006 ▶), motor neurone disease (Puls *et al.*, 2003 ▶), cancer (McClatchey, 2003 ▶) and several myopathies (Oldfors *et al.*, 2004 ▶). Furthermore, the associative properties of coiled coils are exploited in biotechnology for the bio­fabrication of self-assembled and bioactive polymeric materials (Woolfson, 2010 ▶; Pechar & Pola, 2013 ▶). Fields of application include drug delivery, synthetic matrices for cell growth and differentiation, biosensors and antigen-display particles for vaccination (Apostolovic *et al.*, 2010 ▶). In brief, coiled-coil folds are of substantial physiological, biomedical and biotechnological significance, their structural characterization underpinning the rapid development of these fields.

Notably, the apparent simplicity of the coiled-coil architecture does not translate into crystallographic tractability (Franke *et al.*, 2011 ▶; Blocquel *et al.*, 2014 ▶). This is owing to several factors that work in combination to hamper the phasing of these proteins. Firstly, coiled-coil proteins are filamentous in nature and their highly elongated shapes can lead to unusual, tightly packed crystalline lattices in which molecules are laterally associated, resulting in minimal interstitial bulk solvent (see, for example, Blocquel *et al.*, 2014 ▶; Garcia *et al.*, 2004 ▶; Urzhumtsev *et al.*, 2008 ▶). This complicates the separation of self and cross Patterson vectors in molecular replacement (MR; Evans & McCoy, 2008 ▶). Secondly, coiled-coil folds can exhibit notable levels of diversification, displaying both distinct superhelical parameters and local helical distortions caused by deviations from the idealized heptad repeat (*e.g.* stutters; Lupas & Gruber, 2005 ▶). Deceptively subtle, these helical and superhelical irregularities can cause long-range deviations in the fold that trouble the MR method. Finally, MR approaches are significantly hampered by the frequency with which coiled-coil folds differ from expectations, with the assembly of the constituent chains being highly sensitive to experimental conditions, such as the crystallization medium and construct design. This can drastically and unpredictably alter their self-association and thereby the overall fold (see, for example, Kapinos *et al.*, 2011 ▶; Franke *et al.*, 2014 ▶). Thus, even when ultimately successful, MR of coiled-coil proteins is rarely straightforward, often resulting from bespoke and time-consuming screens of potential search models (Communie *et al.*, 2013 ▶; Howard *et al.*, 2007 ▶; Li *et al.*, 2002 ▶). The alternative approach of phasing using MAD data can be hampered by the generally low frequency of methionine and cysteine residues in repetitive coiled-coil sequences.

Recent years have seen the emergence of innovative MR methods that aim to address cases where traditional, homology-based search models are not available. Prominent among these are *AMPLE* (Bibby *et al.*, 2012 ▶, 2013 ▶), which clusters and truncates *ab initio* protein structure predictions to derive ensemble search models of a wide range of sizes, and *ARCIMBOLDO* (Rodríguez *et al.*, 2009 ▶, 2012 ▶), which employs small ideal model fragments, most often α-helices of 10–14 residues, as search models. Each method has seen recent success with coiled-coil structures (Franke *et al.*, 2014 ▶; Bruhn *et al.*, 2014 ▶).

*AMPLE* can generate search models for MR from computationally cheap, low-resolution *Rosetta* *ab initio* predictions (decoys) of individual protein chains (Bibby *et al.*, 2012 ▶). 1000 decoys are generated and clustered on structural similarity. Up to 200 decoys from the largest cluster are then truncated at 20 different levels of severity, based on inter-decoy structural variance, to generate a pool of truncated ensembles, which are subclustered under three different radius thresholds (1, 2 or 3 Å). Up to 30 structures of each subcluster are structurally aligned and are then converted into ensemble search models to trial through three different side-chain treatments: polyalanine, where all of the side chains are truncated at their C^β^ atom; reliable side chains, where only those side chains that are usually well modelled (Shapovalov & Dunbrack, 2007 ▶) are kept; and all-atom, where all side-chains are kept (see §[Sec sec2]2). The number of ensemble search models that are created, and the number of individual conformers that each ensemble contains, will vary depending on the structural diversity amongst the initial decoys and the diversity amongst the models following truncation. Ensembles are processed with *MrBUMP* (Keegan & Winn, 2008 ▶), which runs MR using *Phaser* (Storoni *et al.*, 2004 ▶; McCoy *et al.*, 2005 ▶, 2007 ▶), main-chain tracing by *SHELXE* (Sheldrick, 2008 ▶; Usón *et al.*, 2007 ▶; Thorn & Sheldrick, 2013 ▶) and automatic rebuilding of the *SHELXE* trace with *ARP*/*wARP* (Cohen *et al.*, 2008 ▶; Langer *et al.*, 2008 ▶) or *Buccaneer* (Cowtan, 2006 ▶). Not all *Phaser* jobs return MR solutions, but a crucial part of the pipeline is that every putatively MR-positioned model produced by *Phaser* is submitted blindly to *SHELXE* regardless of whether the statistics indicate that the job has succeeded. A study of 295 test cases comprising small proteins with <120 residues and resolution better than 2.2 Å showed that this approach was very successful, solving 43% of all protein targets in the set and, specifically, 80% of all-α proteins (Bibby *et al.*, 2012 ▶). The time taken to process a given target with *AMPLE* is variable, depending on the size of the modelled chain, the resolution of the diffraction data, the number of ensembles generated and the relative proportion of successful ensembles. Average runtimes are of the order of 24 h on a single CPU, although as *AMPLE* is extensively parallelized, runtimes are typically considerably less than this on a modern multi-core desktop computer.

Encouraged by the high success rate of *AMPLE* on all-α proteins, we embarked here on a study of the application of *AMPLE* to coiled-coil targets and related α-fibrillar folds. These remain challenging for MR phasing, and approximately half of the test cases used here were originally solved with experimental phasing. We found that *AMPLE* could solve most of the test cases (80%), including proteins up to 253 residues in length and examples with resolution data as low as 2.9 Å. Importantly, the process did not require any *a priori* knowledge of the overall fold arrangement or assembly mode of the protein target. Thus, we propose *AMPLE* as a generic tool for the phasing of challenging α-helical protein folds.

## 2. Methods   

### 2.1. Selection of test cases   

A test set of coiled-coil crystal structures was obtained by a search of the PDB (Rose *et al.*, 2013 ▶) for structures with >70% helical content and diffraction data to better than 3 Å resolution containing 1–4 helices and sharing no more than 50% sequence identity. Manual removal of globular proteins resulted in 94 crystal structures (Supplementary Table S1). This set still contains several instances (detailed in Supplementary Table S1) where multiple targets are derived from the same protein or from clearly homologous proteins, often corresponding to overlapping fragments or subfragments. Such targets are common in coiled-coil protein research, where long coiled-coil domains are split into shorter sections for structural work. These were not eliminated by the PDB clustering algorithm for 50% identity redundancy removal since it requires that the alignment must cover at least 90% of the length of both sequences. We chose to retain all of these cases since it is common for coiled-coil substructures to pack differently to their parents (intramolecularly and intermolecularly) so that they should not necessarily be considered as redundant in terms of their crystal structure solution. Of the set of 94 structures, 50 were originally solved by MR and 44 by experimental methods.

### 2.2. *AMPLE* algorithm   

*AMPLE* (Bibby *et al.*, 2012 ▶) was used to generate 1000 cheap *ab initio* predictions (decoys) using *Rosetta* (Simons *et al.*, 1997 ▶, 1999 ▶, 2001 ▶), which were clustered by r.m.s.d. using *SPICKER* (Zhang & Skolnick, 2004 ▶). The appearance of large clusters is considered to be an indication of reliable modelling, so up to 200 decoys from the largest cluster were selected and the structural variation amongst the aligned C^α^ atoms was calculated using *THESEUS* (Theobald & Wuttke, 2006 ▶), allowing the residues to be grouped according to their structural similarity. Structural variance along the chain predicts deviation from the true structure (Bibby *et al.*, 2012 ▶; Qian *et al.*, 2007 ▶), so *AMPLE* constructed a range of search models by pruning back residues at a series of 20 variance levels (truncation thresholds). This generated a pool of truncated and fragmented models that served as the basis for the assembly of search ensembles.

The models under each truncation level were structurally clustered again in a ‘subclustering step’. The centroid representative model of each truncation step served as the basis for the assembly of three increasingly diverse clusters containing structures superimposable on the centroid with overall C^α^ r.m.s.d. values within 1, 2 or 3 Å using *MaxCluster* (http://www.sbg.bio.ic.ac.uk/~maxcluster). These clusters were limited to at most 30 structures and were structurally aligned with *THESEUS* to create an ensemble.

To generate the final search models, these ensembles were subjected to three different side-chain treatments: polyalanine, where all the side chains were stripped back to C^β^; reliable side chains, where only those side chains that are usually well modelled (Shapovalov & Dunbrack, 2007 ▶) were kept, with others being stripped back to alanine; and all-atom, where all side chains were kept.

The ensemble search models were processed with *MrBUMP* (Keegan & Winn, 2008 ▶), which performed MR using *Phaser* (Storoni *et al.*, 2004 ▶; McCoy *et al.*, 2005 ▶; 2007 ▶), main-chain tracing by *SHELXE* (Sheldrick, 2008 ▶; Usón *et al.*, 2007 ▶; Thorn & Sheldrick, 2013 ▶) and automatic rebuilding of the *SHELXE* trace with *ARP*/*wARP* (Cohen *et al.*, 2008 ▶; Langer *et al.*, 2008 ▶) and *Buccaneer* (Cowtan, 2006 ▶).

### 2.3. Running the test cases   

*AMPLE* v.0.1.0 (Bibby *et al.*, 2012 ▶) in the *CCP*4 suite v.6.4.0-008 (Winn *et al.*, 2011 ▶) was used for this study. The study excluded fragments homologous to the target from the *ab initio* model building in order to treat each case as if it were a novel fold. *REFORIGIN* (Winn *et al.*, 2011 ▶) was used to assess the accuracy with which search models were placed with respect to the crystal structure. However, this tool captures only the in-register placements that lie at the heart of conventional MR. In this work, it rapidly became clear that out-of-register overlaps between search models and crystal structures could contribute partially or fully to successful structure solution. We therefore developed the residue-independent overlap (RIO) score to quantify these successes. The RIO score counts the number of C^α^ atoms in the MR result that are within 1.5 Å of a C^α^ atom in the crystal structure (regardless of how the C^α^ atoms are related in sequence), including only stretches where at least three consecutive C^α^ atoms of the MR result overlie matching C^α^ atoms in the deposited structure. This quantifies how well the MR result maps onto the crystal structure, without making any assumptions about the correctness of the model or the placement with respect to register. The total RIO score is composed of an in-register (*i.e.* residues in the placed MR search model correctly overlay their counterparts in the crystal structure) RIO_in component and a second RIO_out component summing out-of-register overlays. Scripts for calculating RIO scores are available on request from the authors: they require both *CCP*4 and *PHENIX* (Adams *et al.*, 2010 ▶) installations. A detailed description of the RIO score calculation pipeline is included in the Supporting Information.

## 3. Results   

### 3.1. Percentage of coiled-coil structures solved using stringent success criteria   

To evaluate the performance of the *AMPLE* algorithm on coiled-coil-like folds, a large and diverse test set of 94 structures (Supplementary Table S1) was selected from the PDB and structure solution was attempted. The 94 targets generated 15 244 search ensembles, with an average of 162 ensembles per target (varying from 18 for PDB entry 1kyc to 297 for PDB entry 3azd). Under normal usage, *AMPLE* would stop as soon as a successful structure solution was produced, using well established statistics from *SHELXE* to detect success, but here all search models were processed in order to assess their performance. The established measure for success of MR with *SHELXE* main-chain tracing and electron-density modification is a correlation coefficient (CC) score of ≥25 and an average traced chain length of ≥10 residues. The generally accepted operating parameters for *SHELXE* include a resolution better than around 2.4 Å. As the targets we examined extended to resolutions as low as 2.91 Å, we tightened our success criteria by requiring that the *SHELXE* trace could be rebuilt by either *Buccaneer* (Cowtan, 2006 ▶) or *ARP*/*wARP* (Cohen *et al.*, 2008 ▶; Langer *et al.*, 2008 ▶) with a resulting *R*_free_ value of ≤0.45. By these criteria, 64 of 94 targets (68%) were successful in a single run of *AMPLE*.

Since each *AMPLE* run builds a specific set of *Rosetta* decoys, which although similar between runs differ in their details, there is an element of chance in structure solution with *AMPLE*: a few borderline cases will solve in one run but not in another. Thus, targets that did not solve by the above criteria in the first run were run twice more. These runs added nine cases (PDB entries 1mi7, 2bez, 2q5u, 2zzo, 3h00, 3h7z, 3tyy, 3u1a and 3u1c) to the tally of successes. Finally, since successful MR placements may not immediately be refinable to the *R*_free_ criterion, we manually inspected the top model (*i.e.* that with the best *SHELXE* CC score) for targets that had not achieved an *R*_free_ of ≤0.45. For two targets (PDB entries 3bas and 3cvf) density suggested successful solution and, indeed, further cycles of rebuilding in *Buccaneer* lowered the *R*_free_ values to 0.44 and 0.32, respectively. These further cases give a total of 75 successes from 94 targets (80% of the set). Supplementary Table S1 summarizes all MR trials in this work on a per-target basis.

### 3.2. Resolved structures spanned all sizes and resolutions tested   

**Figure 1:**
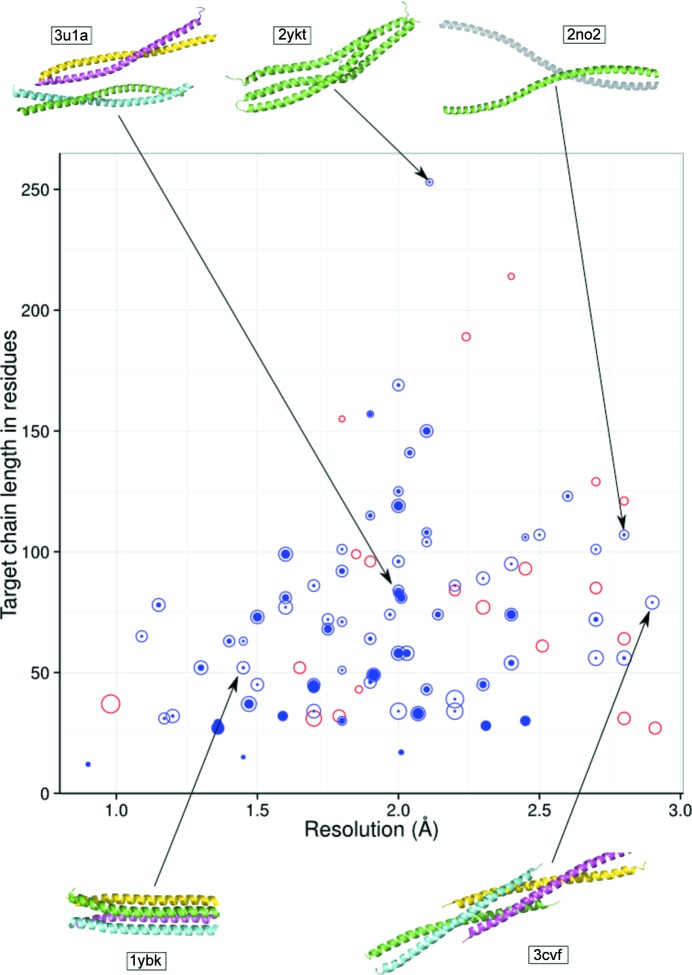
Target success mapped against resolution and target chain length. Each circle represents a target, with the radius of the outer circle proportional to the number of models generated for that target and with the colour indicating whether the target was solved (blue) or not (red). The filled blue circles within the open circles indicate the proportion of successful models. The crystal structures of selected targets are shown, with each chain coloured differently. Asymmetric unit contents are shown except for PDB entry 2n02, where the biological assembly is displayed with the second chain generated by crystallographic symmetry in light grey.

Assessing the characteristics of successfully solved cases, we found that they covered the full range of chain lengths in the test set from the smallest (PDB entry 1byz, a designed protein with 13 residues) to the largest (PDB entry 2ykt, the complex of the N-terminal I-BAR domain of IRSp53 with a bacterial peptide; 253 residues) (Fig. 1 ▶). We believe that 2ykt is the largest protein structure solved to date using *ab initio* predictions for MR phasing.

Similarly, in terms of asymmetric unit content, success was seen over a wide range (Supplementary Fig. S1), with the largest target being PDB entry 3u1a, with four chains in the asymmetric unit totalling 334 residues. Interestingly, PDB entry 3u1a corresponds to a recently elucidated structure of a fragment of smooth-muscle tropomyosin α that was originally phased using SAD on selenium because conventional MR approaches were unsuccessful (Rao *et al.*, 2012 ▶). We observed that the resolution of the diffraction data also had little impact on the likelihood of success or failure: the mean resolution of the failures was 2.03 Å, while that of the successes was 1.88 Å. The lowest resolution structure (PDB entry 3v86 at 2.91 Å resolution) failed to solve, but three of the lower resolution cases in our set (PDB entries 3cvf at 2.90 Å and 2w6b and 2no2 both at 2.80 Å) all solved; a notable outcome given that *SHELXE*, upon whose results we judge success, is only generally considered to work well up to 2.4 Å resolution and is reported to require better than 2.1 Å resolution data for expansion from small fragments (Thorn & Sheldrick, 2013 ▶). These low-resolution successes are remarkable, in particular PDB entry 2no2, a domain of huntingtin-interacting protein 1 which contains a long, unconventional coiled-coil-like assembly (Fig. 1 ▶) that was originally phased experimentally using MAD. Similar to the other parameters evaluated, the solvent content of crystals of successfully solved cases (mean of 46.6%) was not significantly different from that of the failures (mean of 50.4%). The fact that some targets were solved only with small numbers of search models (*e.g.* PDB entry 1kql solved with only one from a set of 234), demonstrates the value of *AMPLE*’s extensive sampling.

These results indicate that *AMPLE* is an universal tool for the phasing of coiled-coil-like assemblies that can resolve structures over a broad range of sizes, not being limited *a priori* by resolution or crystallographic parameters. Most importantly, *AMPLE* does not require previous knowledge of the arrangement of chains (parallel/antiparallel) or their level of oligomerization and thus can succeed in unconventional cases that remain challenging for classical MR approaches. For example, both of the two unusual right-handed coiled coils in the test set solved, namely the structures of the bacterial surface-layer protein tetrabrachion (PDB entry 1ybk; see Fig. 1 ▶) and the vasodilator-stimulated phosphoprotein tetramerization domain (PDB entry 1usd). Also successful were the two other structures in the set containing both right-handed and left-handed coiled coils [human lamin (PDB entry 2xv5) and bacterial autotransporter segments (PDB entry 3h7z)].

### 3.3. Exploiting coiled coils for the structure solution of complexes   

Coiled-coil proteins are often involved in the formation of protein complexes owing to their biological roles in scaffolding, transcription and cell signalling (Burkhard *et al.*, 2001 ▶; Rose *et al.*, 2005 ▶). The conspicuous success of *AMPLE* in solving coiled-coil targets led us to speculate that sufficient phasing information could be obtained through MR with *AMPLE* applied to coiled-coil components of complexes, to then permit the tracing of protein or polynucleotide partners in biological assemblies. Phasing with *AMPLE* and coiled-coil subunit sequences was attempted for two such complexes (PDB entries 1x79 and 1h8a) identified using the SCOP database (Andreeva *et al.*, 2008 ▶).

**Figure 2:**
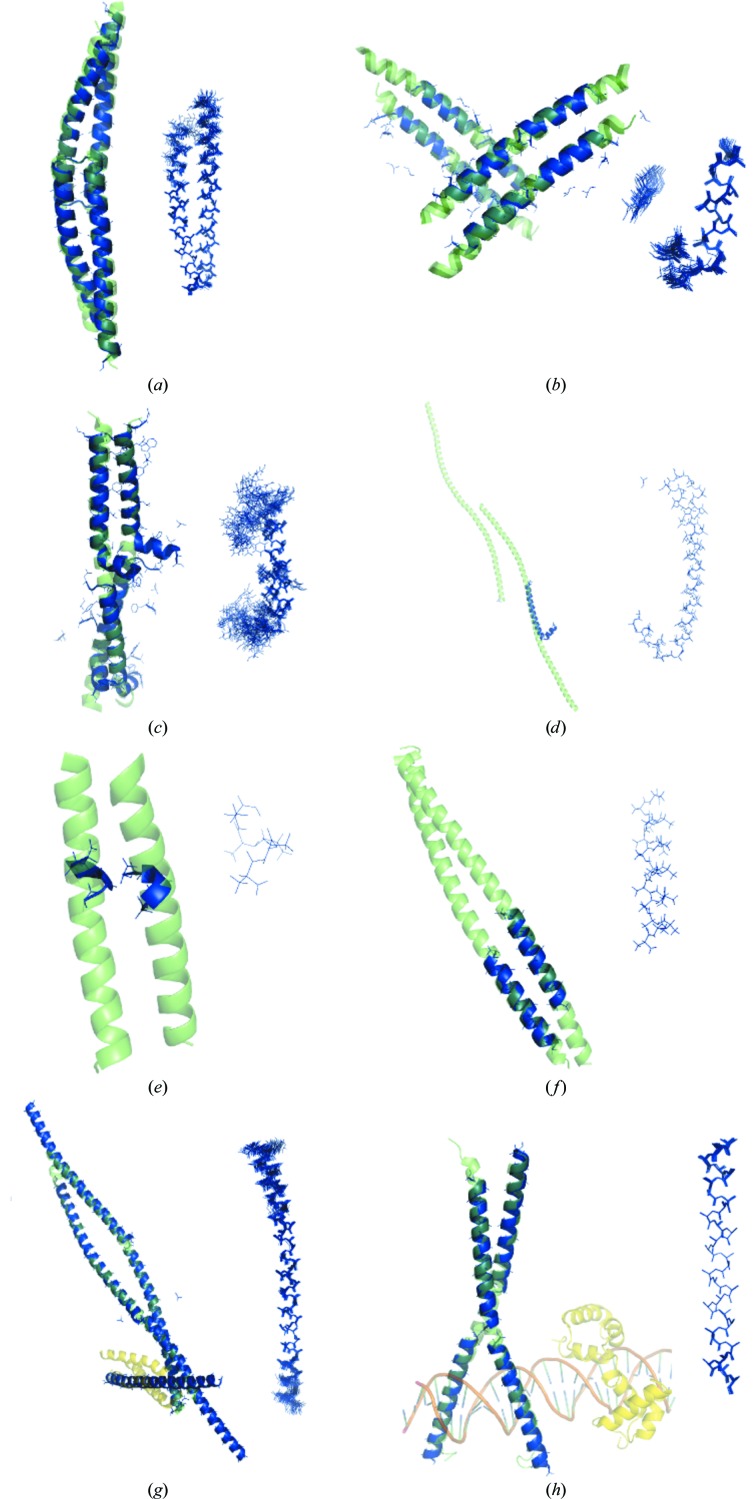
Illustrative examples of successful coiled-coil structure solution with *AMPLE*. In each case, the target chain of the crystal structure is shown on the left as a green cartoon, MR-placed model(s) as blue lines and (where appropriate) cartoons, and the ensemble search model is displayed on the right in blue. MR-placed model ensembles (*a*–*d*, *g*–*h*) are represented here, for clarity, by their first member. (*a*) In-register placement (*i.e.* the sequence of the search model correctly aligns with that of the substrate) of two copies of a mildly truncated centroid structure as a search model solves the coiled-coil domain structure of the *Sin nombre virus* nucleocapsid protein (PDB entry 2ic9). (*b*) Out-of-register placement (the backbone structures of the search model and target coincide closely, but their sequences do not match) of eight copies of a heavily truncated search-model ensemble with polyalanine side-chain treatment solves a coiled-coil fragment from the HIV-1 protein gp41 (PDB entry 3h00). (*c*) Four copies of an ensemble with reliable side-chain treatment solves the coiled-coil domain structure from the replication regulator geminin (PDB entry 1uii). (*d*) A heavily truncated polyalanine search model solves the structure of adhesin UspA1 (PDB entry 2qih). (*e*) Two copies of a five-residue ideal polyalanine helix solved a *de novo*-designed assembly protein (PDB entry 3s0r; 2.45 Å). (*f*) Two copies of a 20-residue ideal polyalanine helix solved a dynamin adaptor protein (PDB entry 2xu6; 2.7 Å). (*g*) Five copies of a mildly truncated polyalanine search model ensemble solved the complex of the GGA1 GAT domain (yellow) with the GAT-binding domain of rabaptin 5 (green) (PDB entry 1x79; 2.4 Å). (*h*) Four copies of a polyalanine search model ensemble solved a transcription-regulation complex (PDB entry 1h8a; 2.23 Å) containing a coiled-coil domain (green), an additional helical protein (yellow) and duplex DNA (brown).

PDB entry 1x79 contains 322 residues and comprises a dimeric coiled-coil domain in complex with a small, helical GAT domain (Fig. 2 ▶ *g*). The structure solved with an ensemble search model of the coiled-coil chain, 112 residues in length, ultimately tracing and refining at 2.41 Å resolution to *R* = 0.25, *R*_free_ = 0.29 after *ARP*/*wARP*. The backbone structure was accurately traced in the final result, although there were errors in the assignment of the sequence to the trace. PDB entry 1h8a contains 284 residues, a dimeric coiled-coil domain (2 × 78 residues) from CCAAT/enhancer-binding protein β and a Myb DNA-binding domain, in complex with a 26-base-pair DNA duplex. The DNA contributes about a third of the scattering matter of the crystal. Nevertheless, search models deriving from predictions of the coiled-coil sequence solved the structure. Taking the *AMPLE*-produced *SHELXE* C^α^ trace as a starting point, a rough initial model for the target could be built using a combination of *Buccaneer* for the protein molecules and *Nautilus* (Cowtan, 2012 ▶) for the DNA (Supplementary Fig. S2*a*). Phases from this initial model were then further improved in *SHELXE*. With these improved phases a more complete model could be built with *Buccaneer*/*Nautilus*, achieving *R* = 0.39 and *R*_free_ = 0.42 at 2.23 Å resolution (Supplementary Fig. S2*b*).

### 3.4. Successful search models   

In order to understand the successes of *AMPLE*, we examined the search models and their respective performance. Analysis of the length distributions of the successful search models (Supplementary Fig. 3) indicated that search models of all lengths were capable of solving structures, but the majority tended to be relatively short, approximately 20–30 residues in length.

Remarkably, some successful search models contained only minimal portions of the target. The structure of a phospholamban variant (PDB entry 1yod) was solved with a search ensemble with the smallest number of residues, five, representing 16% of the target chain length. This reiterates the value of sampling over a range of truncations from mild to substantial. Some of these small fragments were successfully expanded in *SHELXE* at resolutions worse than the 2.1 Å currently considered to be required (Thorn & Sheldrick, 2013 ▶); for example, the structure of a designed protein (PDB entry 3s0r with a resolution of 2.45 Å) solved with an ensemble with nine residues. *AMPLE*’s strategy of submitting all *Phaser* solutions to *SHELXE* regardless of the *Phaser* statistics was also reinforced in the current work, as can be seen in Supplementary Fig. S4, where successful solutions were found with *Phaser* LLG scores as low as −833 and TFZ scores as low as 2.1.

Probing the accuracy of the search-model placement by *Phaser* using the *CCP*4 tool *REFORIGIN* showed that many search models succeeded despite the r.m.s.d. of the placed model to the crystal structure indicating that the model had been placed ‘incorrectly’ (Supplementary Fig. S5). *REF­ORIGIN* assumes a correspondence in residue numbering between the placed model and the crystal structure, but the examination of a number of successful solutions demonstrated that many derived from small helical fragments that had been placed out of register, *i.e.* their backbone overlaid productively with the crystal structure but the sequence of the fragment did not match that of the region upon which it was placed. To quantify these placements, we developed the RIO score (see §[Sec sec2]2) which measures accurate backbone placement based on C^α^—C^α^ distances between the positioned search model and the crystal structure. The total RIO score sums RIO_in (conventional, sequence-matching in-register placements) and RIO_out (out-of-register placements) components. An analysis of the successful solutions revealed that the majority derived from these out-of-register alignments. This suggests that it is the helical main-chain path that dominates the MR outcome, with the contribution of the side chains being dispensable. Fig. 2 ▶ shows a representative selection of solutions ranging from largely complete in-register solutions to small out-of-register solutions. Consistent with the predominance of out-of-register success, polyalanine search models were the most successful overall among the different side-chain treatments (Supplementary Fig. S6), with their lack of side chains presumably rendering them less sensitive to register errors.

### 3.5. Solving structures with ideal polyalanine helices   

The success of short, out-of-register helical fragments led us to question the extent to which the success of *AMPLE* derived from its ability to select promising fragments of the *Rosetta* decoys and whether these models have advantages over simpler, ideal α-helices. We therefore attempted to solve the original test set using ideal polyalanine α-helices as search models. As the most successful search ensembles were between 20 and 30 residues in length, eight ideal α-helices were generated starting at five residues in length and extending to 40 in five-residue increments, with ideal backbone torsion angles ϕ = −57.8° and ψ = −47.0°.

These simple polyalanine helices solved 52 of the targets (55%), including PDB entry 2qih with 278 residues (two chains) in the asymmetric unit and PDB entry 2w6b with a resolution of 2.8 Å (Supplementary Fig. S7). The success covered almost the entire breadth of the test set. Figs. 2 ▶(*e*) and 2 ▶(*f*) show examples of differently sized polyalanine helices that solved structures with a resolution poorer than 2.4 Å. However, *AMPLE* with *ab initio* prediction-derived search models performed considerably better, solving an additional 23 targets. In particular, the success of ideal helices in the lower resolution range (data to worse than 2.5 Å) was notably lower than with ensembles: only two targets in this set of 15 solved with ideal helices. Considering the effort often devoted to preparing search models for MR, it is surprising to discover that many coiled-coil structures, including those with long chains and limited resolution, could be solved solely with these simple helical models. Despite the polyalanine helices faring relatively well, the results confirm the added value of the *AMPLE* process.

### 3.6. Ensembles as best-suited MR search models   

The two primary factors that differentiate *AMPLE*’s approach from ideal helices are the modelling by *Rosetta* and the creation of ensembles rather than single-structure search models. In order to disentangle the effect of the ensembling, we re-tested the original set using the first model from each ensemble. As *AMPLE* builds ensembles by subclustering the truncated decoys around a first structure, the latter roughly corresponds to the centroid of the ensemble. We therefore compared the performance of the centroid single structure with its parent ensemble calculated using a 1 Å subclustering radius (the subclustering radius that performed best; Supplementary Fig. S8).

**Figure 3:**
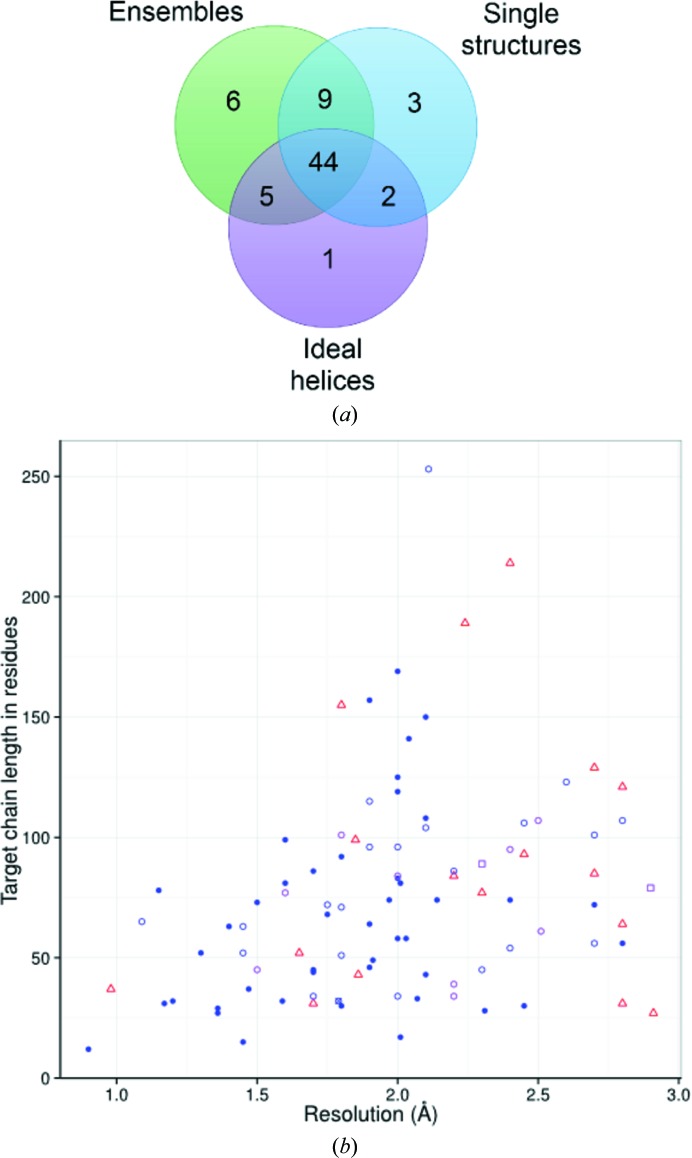
*Ab initio* model-derived search-model ensembles solve more coiled-coil targets than either single structures or ideal helices. (*a*) Overall successes with a single run of ensemble (green), single-structure (blue) or ideal polyalanine helix (purple) search models. A single structure, PDB entry 3h00, solved with ideal helices that did not solve with a single run of *ab initio* prediction-derived search models. (*b*) Detailed breakdown of targets solved with ensembles, single structures and/or polyalanine helices. Red triangles, failures; filled blue circles, solved with each search model (ensemble, single structure and helices); empty blue circles, solved with one or more of the above; blue circle with a cross, only solved with the single-structure run (PDB entry 4dzk); purple circles, solved with a re-run of the ensemble (PDB entries 1mi7, 2bez, 2q5u, 2zzo, 3h00, 3h7z, 3tyy, 3u1a and 3u1c); purple square, manual inspection indicated success (PDB entries 3bas and 3cvf).

The results showed that ensembles are somewhat more successful than single centroid structures, having solved 66 targets here in a single run against the latter’s 58 (Supplementary Fig. S9). Despite the overall greater success of ensembles, five targets (PDB entries 2q5u, 3q8t, 3tyy, 3u1c and 4dzk) were solved with centroid search models but not with a single run of the ensembles (Fig. 3 ▶). Of these, four were solved in one of the two subsequent reruns of the ensembles, but PDB entry 4dzk was only solved with the single-structure search model. This suggests that ensembling, a key characteristic of *AMPLE*, is generally an efficient strategy for gainfully combining information from a number of predictions into a single search model. However, in a small minority of cases the potential success that would be achieved with a single structure is masked by the ensembling.

## 4. Discussion   

MR is an increasingly popular route to protein structure solution. Historically, it has largely been confined to cases of recognisable homology between the unknown target and an already determined structure: the latter, or a homology model of the target based upon it, served as the search model. More recently, two relatively niche approaches have emerged to eliminate this restriction of homology, but each generally brings very significant CPU demands. The first is to use *ab initio* modelling to derive search models for targets that lack recognisable homologues in the PDB. Pioneering work showed that this could succeed for small, globular proteins (Das & Baker, 2009 ▶; Qian *et al.*, 2007 ▶), but the very computationally intensive, all-atom protocols that it used place the method out of reach of typical crystallography laboratories. The second approach, pioneered by *ARCIMBOLDO* (Rodríguez *et al.*, 2009 ▶, 2012 ▶), uses small search models, commonly ideal α-helices, to represent portions of the unknown structure that can be reliably predicted, even in the absence of a known fold. This method has achieved some conspicuous successes, but again often brings prohibitive CPU demands. Our previous work with *AMPLE* showed that rapidly obtained *ab initio* structure predictions could be assembled, through clustering and truncation, into successful search ensemble models, where truncation had removed the largest uncertainty in the modelling. Although relatively complete models sometimes succeeded, highly truncated and fragmentary search models were more commonly successful, establishing *AMPLE* as a tool bridging both of the approaches mentioned above.

Emboldened by *AMPLE*’s success with a real-world coiled-coil case (Bruhn *et al.*, 2014 ▶), we here obtained a large and diverse test set of coiled-coil structures and showed that *AMPLE*’s *ab initio* modelling-based approach could solve most of them with only relatively modest CPU demands, in many cases in less than 24 h using a standard multi-core desktop computer. Our comparisons of *AMPLE*’s regular search-model ensembles with single centroid structures and ideal polyalanine helices (Fig. 3 ▶) reveal the contributions to the success of both modelling by *Rosetta* and the ensembling-and-truncation process. In a single *AMPLE* run, the ensembles solved 68% (64 cases) of 15 244 search models, compared with 62% with single structures (58 cases from 5286 search models), endorsing the value of ensembles as search models. Specifically, it is likely here that divergence within the ensemble, even after truncation of the regions predicted to be the least reliable (Bibby *et al.*, 2012 ▶), reflects residual local uncertainty and can be usefully downweighted by the MR procedure. The success rate with a set of ideal polyalanine helices at 55% (52 cases) was surprisingly high but distinctly lower than the modelling-derived search-model sets, underlining the value of employing explicit tertiary-structure prediction. This can be rationalized here by consideration of the variable but generally significant distortions from α-helix ideality found in coiled-coil structures (Lupas & Gruber, 2005 ▶; Strelkov & Burkhard, 2002 ▶). Since the nine-residue fragments that contribute to *Rosetta* model building are long enough to detect the classical coiled-coil heptad repeat, the resulting models will include helices with distortions from ideality reflecting those naturally occurring in the PDB. Given the poor overall quality of the modelling (data not shown), it is unreasonable to expect the decoys produced to faithfully reproduce the fold-specific bends and twists of the target. Nevertheless, by assembling fragments from naturally occurring distorted helices, and favouring structures containing plausible helical packing modes, the *ab initio* modelling provides sets of structures from which usefully non-ideal helical search models suitable for solving coiled-coil structures can be derived. Fig. 2 ▶(*a*), for example, shows the significant α-helical bend seen in one of the successful search models that solved the 2ic9 structure. Although the sequence is obviously required to direct the overall modelling, often resulting in structures containing authentic helical distortions, polyalanine search models proved most successful overall (Supplementary Fig. S6). A polyalanine search model can be placed at many out-of-register locations with equivalent signal-to-noise ratio, whereas out-of-register side chains are likely to add comparatively more noise than the polyalanine counterpart. Thus, we conclude that polyalanine segments have the potential to generate productive matches with more promiscuity than sequence-explicit search models. Finally, the tractability of coiled-coil structures for *ab initio* structure-based MR extends to complex structures (Figs. 2 ▶ *g* and 2 ▶ *h*). Structure solution *via* a coiled-coil component produces phases that are good enough to allow modern automatic rebuilding tools to place other components, potentially even including novel folds and nucleic acids.

In summary, our results suggest that MR with *AMPLE* should now be the structure-solution method of choice for coiled-coil folds or for other challenging α-helical fibrillar proteins. It makes no assumptions about the architecture of the coiled-coil target (working equally well with right-handed coiled coils), functions effectively over a large target-size range and copes with poor resolution diffraction data down to 2.9 Å. In this study, we have not identified specific target features that are deterministic of success or failure: successfully resolved and failing target sets are similar in diffraction resolution, length, number of residues in the asymmetric unit and solvent content. Particularly if higher resolution data are available, the use of *AMPLE*’s inbuilt library of short ideal helices (shortly to be made available in *CCP*4) may offer the most rapid solution, even though the best performance will be obtained using *ab initio* protein structure modelling. The *Rosetta* models used here perform well, but the user may also try *QUARK* models (Xu & Zhang, 2012 ▶, 2013 ▶), now available from a server (http://zhanglab.ccmb.med.umich.edu/QUARK/) in a format accepted by *AMPLE* (Keegan *et al.*, 2015 ▶). Results with coiled-coil complexes suggest that, far from being an undesirable crystallographic complication (Blocquel *et al.*, 2014 ▶), the presence of a coiled coil in a complex may in fact offer a convenient route for phasing.

## 5. Related literature   

The following references are cited in the Supporting Information for this article: Abergel (2013 ▶) and Murshudov *et al.* (2011 ▶).

## Supplementary Material

Supplementary table 1. DOI: 10.1107/S2052252515002080/lz5005sup1.xlsx

Supplementary methods and supplementary Figs. 1-9.. DOI: 10.1107/S2052252515002080/lz5005sup2.pdf
