# Supplementary material for: Routine phasing of coiled-coil protein crystal structures with AMPLE
Source: IUCrJ. 2015 Feb 26;2(Pt 2):198–206. doi: 10.1107/S2052252515002080 (PMC4392414; doi:10.1107/S2052252515002080)
Supplement: Supplementary file 2 [file m-02-00198-sup2.pdf]

IUCrJ

Volume 2 (2015)

Supporting information for article:

Routine phasing of coiled-coil protein crystal structures with AMPLE

Jens M. H. Thomas, Ronan M. Keegan, Jaclyn Bibby, Martyn D. Winn, Olga Mayans and Daniel J. Rigden

Supplementary Information to "Routine phasing of coiled-coil protein crystal structures with AMPLE" by Thomas *et al.*

Supplementary Methods

RIO algorithm

The algorithm for calculating the RIO score is as follows:

- the MR PDB is renumbered to bring the residue numbers between it and the crystal structure into correspondence.
- the MTZ file containing the crystallographic data is parsed to determine the F, SIGFP and FREE column labels.
- a density map for the crystal structure is generated from the crystal structure PDB file and the MTZ file using REFMAC [1]
- the PHENIX [2] tool get_cc_mtz_pdb uses the density map and the MR result to determine the origin of the MR result with respect to the crystal structure.
- the MR result is moved onto the same origin as the crystal structure.
- the MR result and the crystal structure are concatenated into a single PDB file (with the chains renamed in order to distinguish the two).
- CCP4 NCONT is used to count all contacts between the Cα atoms within 1.5Å between the crystal and MR structures.
- the output of NCONT is parsed to determine the RIO score, as well as determining how many Cα atoms are positioned in- and out-of-register.
- optionally the CCP4 tool CSYMMATCH can be used to overlay the chains in the MR result onto the crystal structure providing a visual representation of how well the MR result has worked.

1. Murshudov, G.N. et al. REFMAC5 for the refinement of macromolecular crystal structures. Acta Crystallogr. D Biol. Crystallogr. 67, 355-367 (2011).
2. Adams, P.D. et al. PHENIX: a comprehensive Python-based system for macromolecular structure solution. Acta Crystallogr. D Biol. Crystallogr. 66, 213-221 (2010).

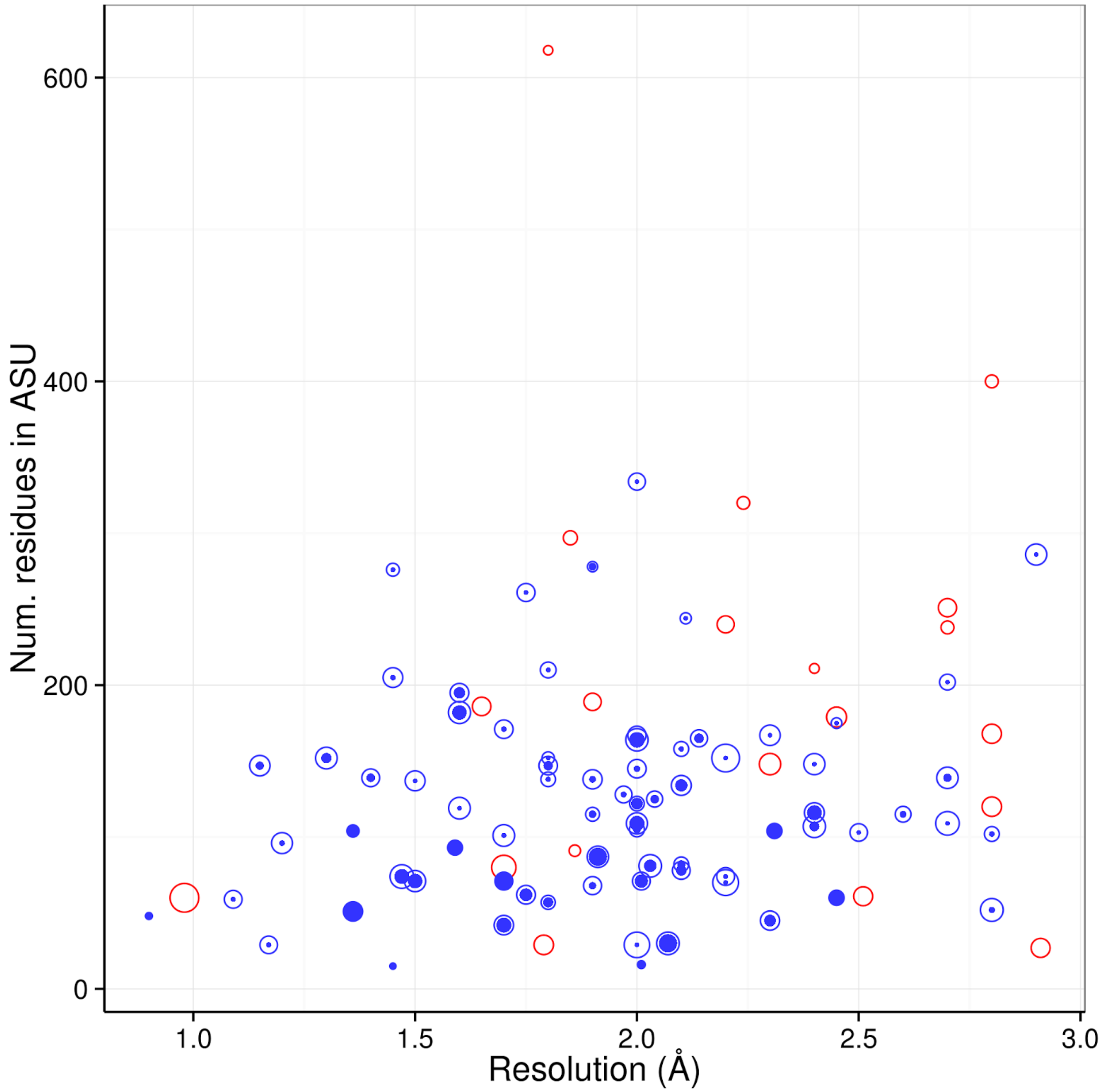

Supplementary Figure S1. **Target success mapped against resolution and the number of residues in the ASU.** Each circle represents a target with the radius of the outer circle proportional to the number of models generated for that target, and the colour indicating whether the target solved (blue) or not (red). The filled blue circles within the open circles indicate the proportion of successful models.

a)

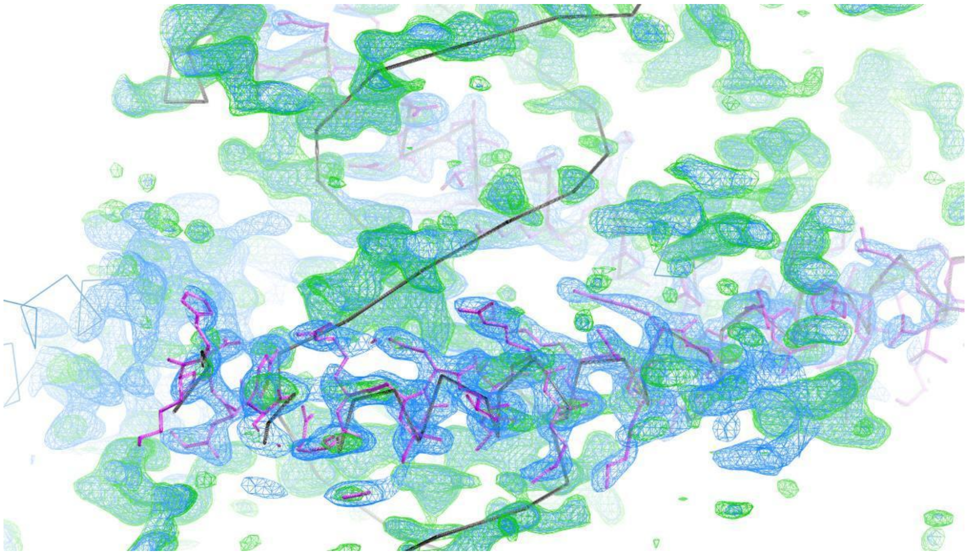

b)

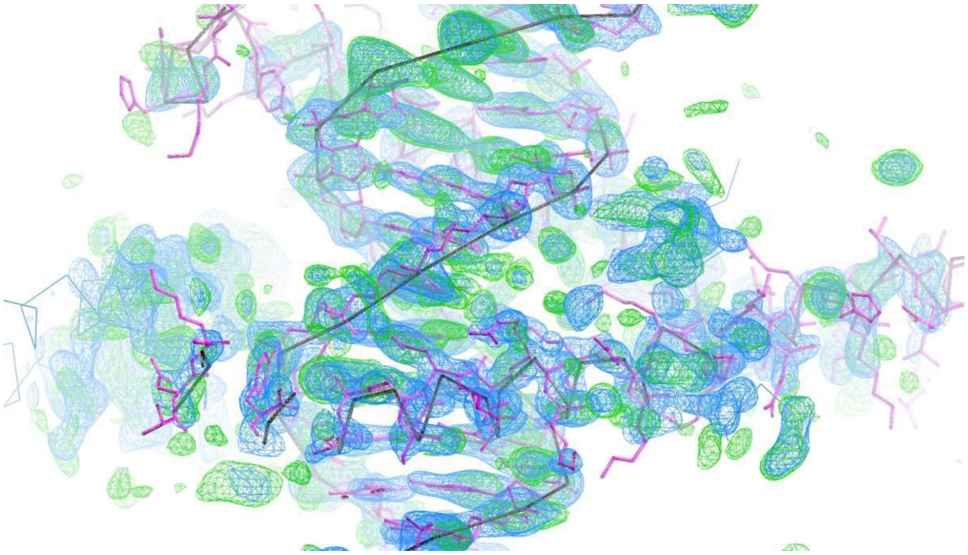

Supplementary Figure S2. **Electron density maps of 1H8A a) immediately post-AMPLE and b) after phase improvement with SHELXE and further model building with Buccaneer/Nautilus (see text for details).** In blue, the $(2F_o\text{-}F_c)\alpha c$ map is contoured at 2σ: positive difference map $(F_o\text{-}F_c)\alpha c$ contoured at 2σ is shown in green. The crystal structure is shown as a ribbon (black with blue used for its symmetry mates) while the structure built at each stage is indicated in magenta. The density in a) supported the correctness of the MR solution, for example revealing density resembling elements of a DNA duplex backbone, later shown (b) to indeed correspond to this missing component.

a)

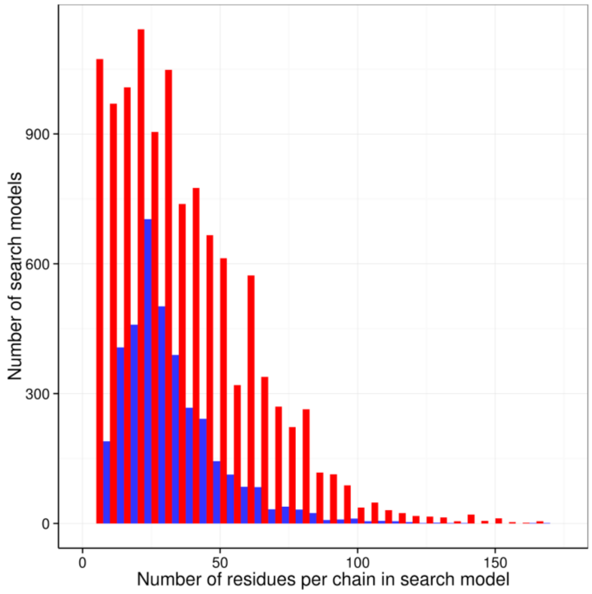

b)

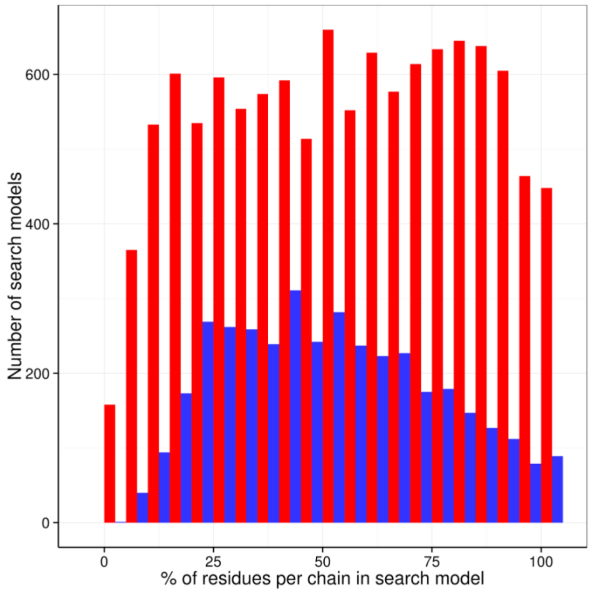

Supplementary Figure S3. **Size distribution of successful or failing search models.** All search models are included, with on average 162 per target. Successful (blue) and failing (red) cases are binned according to a) number of residues per chain in the model and b) residues in the search model as a percentage of the target's chain length. It is worth noting that small fragments, corresponding to highly incomplete search models can be successful: one such example is shown in Fig. 2e.

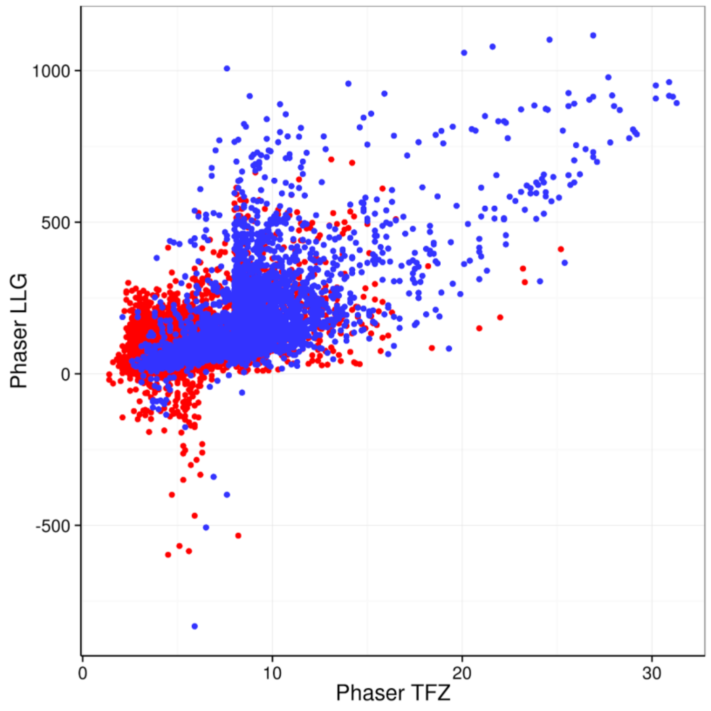

Supplementary Figure S4**. PHASER Log Likelihood Gain score (LLG) against Translation Function Z-score (TFZ) for successful (blue) and failing (red) ensemble search models**.

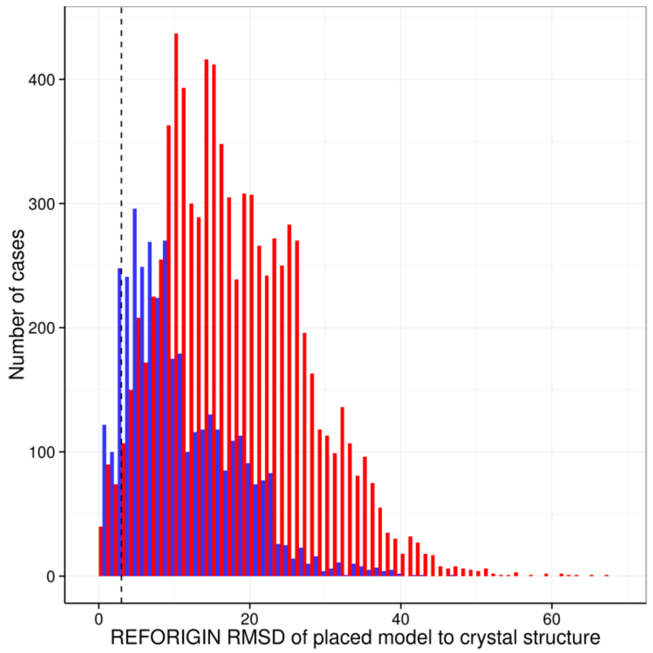

Supplementary Figure S5. **Most successful solutions derive from inaccurate search model placements.** The distributions of REFORIGIN RMSD values for successful (blue) and failing (red) search models are shown. For conventional, homology-based MR, estimates of the maximum allowable divergence between search model and target crystal structure vary [1-3] but we have seen success up to RMSD of 3Å [4], as indicated by the dashed black line.

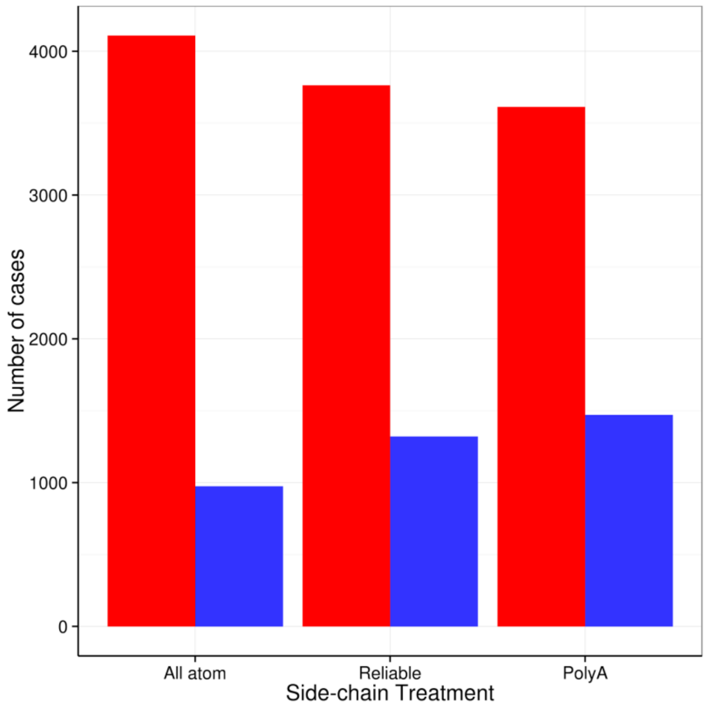

Supplementary Figure S6. **Number of successes (blue) and failures (red) for different ensemble side chain treatments.**

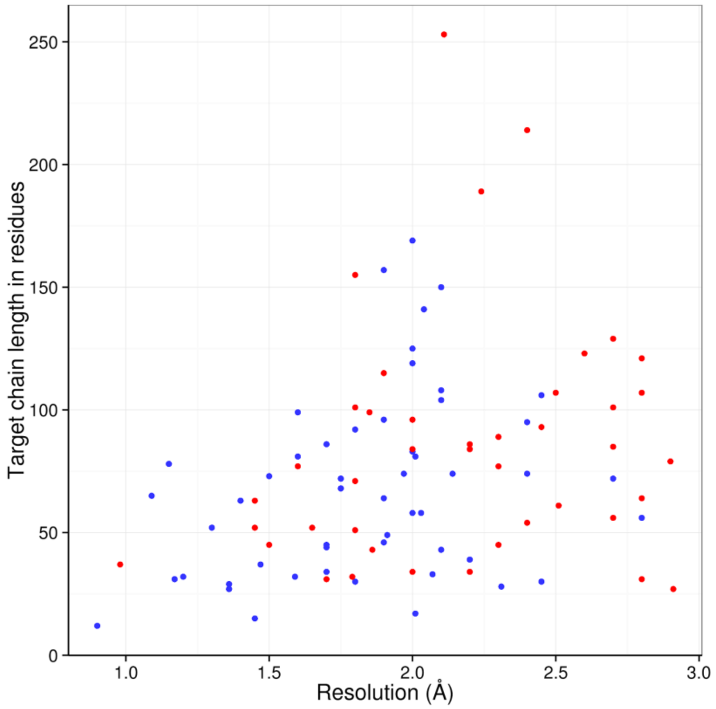

Supplementary Figure S7. **Ideal polyalanine helices solve around half of coiled-coil targets in AMPLE.** Targets are coloured blue (solved) or red (failed) and are plotted by the target chain length and diffraction data resolution.

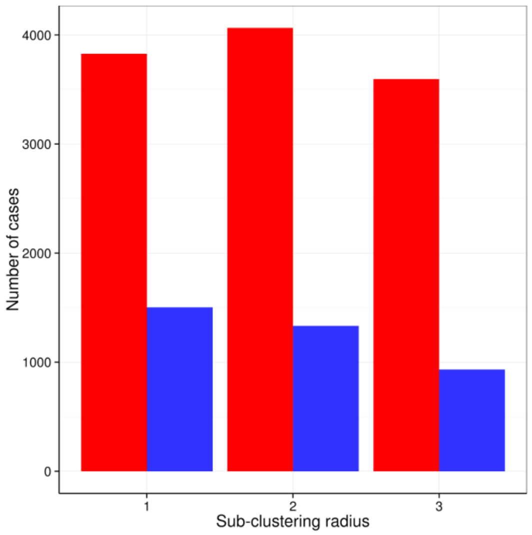

Supplementary Figure S8. **Number of successes (blue) and failures (red) for different ensemble sub-clustering radii.**

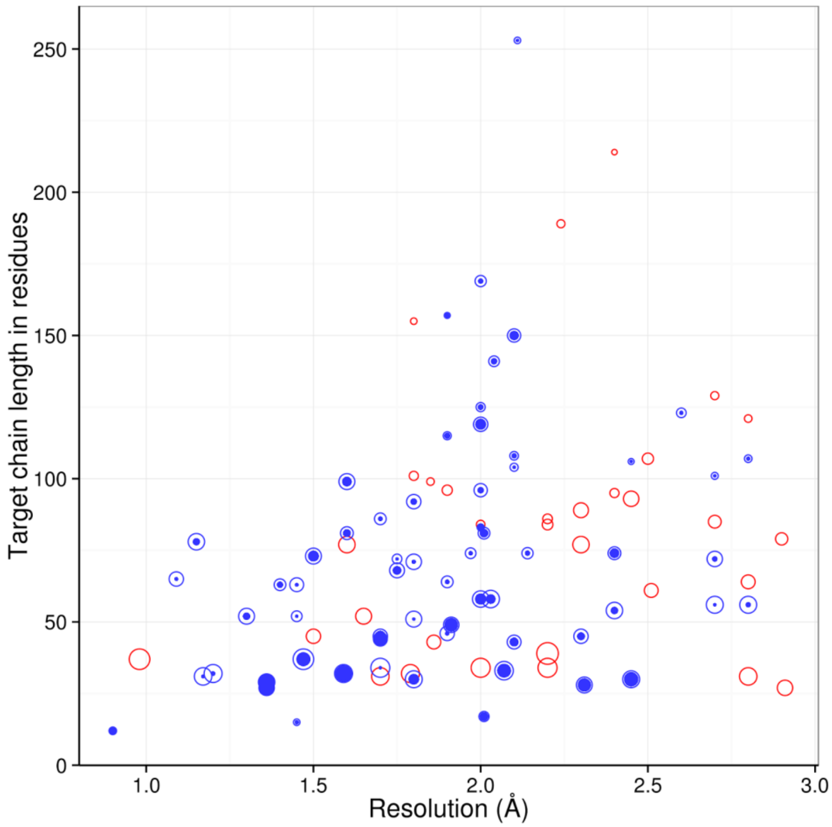

Supplementary Figure S9. **Target success mapped against resolution and target chain length for single-conformer (centroid) search models.** Each circle represents a target with the radius of the outer circle proportional to the number of models generated for that target, and the colour indicating whether the target solved (blue) or not (red). The filled blue circles within the open circles indicate the proportion of successful models.

References to Supplementary Figures

[1] Abergel C. (2013) *Acta Cryst. D***69**, 2167-2173.

[2] http://www.phenix-online.org/documentation/reference/mr_overview.html

[3] http://xray0.princeton.edu/~phil/Facility/Guides/MolecularReplacement.html

[4] Bibby, J., Keegan, R. M., Mayans, O., Winn, M. D. & Rigden, D. J. (2013). *Acta Cryst. D***69,** 2194-2201.
